# Probiotics and Prebiotics as a Strategy for Non-Alcoholic Fatty Liver Disease, a Narrative Review

**DOI:** 10.3390/foods10081719

**Published:** 2021-07-25

**Authors:** Valentina Castillo, Fernanda Figueroa, Karoll González-Pizarro, Paz Jopia, Claudia Ibacache-Quiroga

**Affiliations:** 1 Escuela de Nutrición y Dietética, Facultad de Farmacia, Universidad de Valparaíso, Valparaíso 2360102, Chile; valentina.castillo@alumnos.uv.cl (V.C.); fernanda.figueroaa@alumnos.uv.cl (F.F.); karoll.gonzalez@uv.cl (K.G.-P.); 2 Centro de Micro-Bioinnovación, Universidad de Valparaíso, Valparaíso 2360102, Chile; paz.jopia@uv.cl

**Keywords:** non-alcoholic fatty liver disease (NAFLD), gut microbiota, prebiotics, probiotics, synbiotics

## Abstract

Non-alcoholic fatty liver disease (NAFLD) is a chronic non-communicable disease, with a prevalence of 25% worldwide. This pathology is a multifactorial illness, and is associated with different risks factors, including hypertension, hyperglycemia, dyslipidemia, and obesity. Beside these predisposing features, NAFLD has been related to changes in the microbiota, which favor the disease progression. In this context, the modulation of the gut microbiota has emerged as a new therapeutic target for the prophylaxis and treatment of NAFLD. This review describes the changes in the gut microbiota associated with NAFLD and the effect of probiotics, prebiotics, and synbiotics on the gut microbiota, liver damage, anthropometric parameters, blood lipids, inflammation markers and insulin resistance in these patients.

## 1. Introduction

Noncommunicable diseases (NCDs) are considered a major public health issue worldwide, causing near 41 million deaths per year, which corresponds to 71% of total annual deaths [1]. The main NCDs are cardiovascular diseases, cancer, chronic respiratory diseases, and diabetes mellitus, whose risk factors include the use of tobacco, alcohol consumption, physical inactivity, and a diet high in fat, sodium, and refined sugars. These are predisposing factors to metabolic disorders such as hypertension, hyperglycemia, dyslipidemia, and obesity. Currently, NCDs represent a cost of more than US $11.2 billion in the implementation of interventions to reduce their high impact [2].

NCDs-associated metabolic imbalance is related to the development of other pathologies, whose main risk factors are obesity and diabetes mellitus, which play a fundamental role in their pathogenesis [3], including non-alcoholic fatty liver disease (NAFLD). NAFLD has been acknowledged as the hepatic manifestation of metabolic syndrome (MetS), characterized by central obesity, hypertension, dyslipidemia, and diabetes mellitus [4]. This disease is defined as the accumulation of ≥5% of fat in the liver in the absence of other causes, such as excessive alcohol consumption, viral infections, drugs, and autoimmune diseases [5]. It ranges from hepatic steatosis to non-alcoholic steatohepatitis (NASH), which can progress to cirrhosis, liver failure, and, less commonly, cancer [3]. It has become the most common chronic liver disease [6], with a 25% prevalence worldwide, being higher in the Middle East (31.8%) and South America (30.5%) [7]. Additionally, it occurs more in men (30–40%) than women (15 to 20%) [3,8].

**Figure 1:**
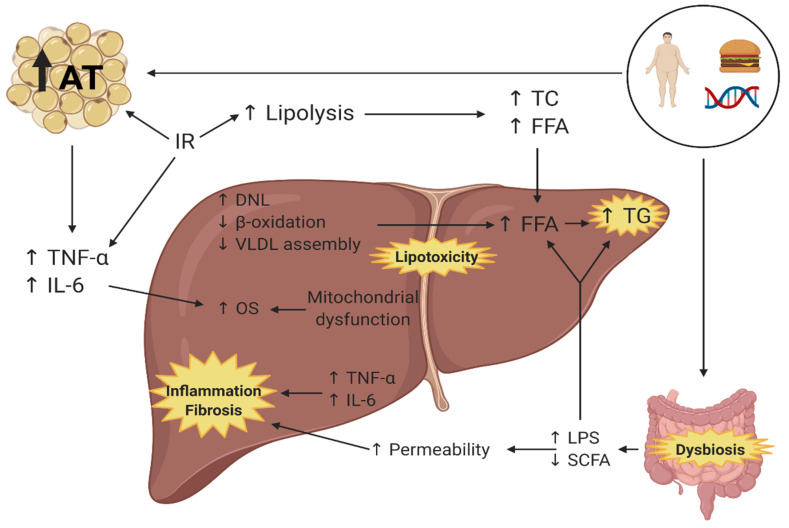
Pathophysiology of non-alcoholic fatty liver disease [9,10,11,12]. AT: Adipose tissue, IR: Insulin resistance, TNF-α: Tumor necrosis factor alpha, IL-6: Interleukin 6, OS: Oxidative stress, DNL: De novo lipogenesis, VLDL: very low-density lipoproteins, FFA: Free fatty acids, TG: Triglycerides, TC: Total cholesterol, LPS: Lipopolysaccharides, SCFA: short chain fatty acids. Created with BioRender.com. Accessed date: 16 April 2021.

The pathophysiology of NAFLD is related to multiple simultaneous factors (theory of multiple impact), such as the genetic background, the environmental conditions and diet, generating insulin resistance (IR), dysfunction of the adipose tissue, obesity, and changes in the gut microbiota [9,10]. These factors produce a disbalance in the acquisition and elimination of hepatic lipids through increased lipolysis and lipogenesis, rising the circulating concentration and uptake of free fatty acids (FFA). This increases the production of pro-inflammatory cytokines and decreases beta-oxidation and the assembly and release of very low-density lipoproteins (VLDL) to circulation [10,11]. In addition to the above, during the past years, alterations in the composition of the gut microbiota, called gut dysbiosis, have been associated with the development of NAFLD. In these patients, it contributes to its physiopathology [12] (Figure 1).

The gut microbiota (GM) is the group of microorganisms that inhabits the gastrointestinal tract, which includes bacteria, fungi, parasites, and viruses [13]. The total number of bacteria in the GM is of 10^14^ UFC [14], a count that varies among the different regions of the gastrointestinal tract, being more abundant in the colon with an estimated count of 10^11^ to 10^12^ of bacterial cells per milliliter [15]. This microbial community is directly involved in the maintenance of the intestinal epithelium integrity, the protection against pathogens, the regulation of the host immunity, the energy harvest, and the regulation of metabolism, among other functions [16]. The composition of the GM is modulated by multiple factors, such as diet, geographic location, and medication consumption [17], being unique in each person. In healthy adults, the GM is mainly composed of Firmicutes and Bacteroidetes phyla, followed by Actinobacteria and Proteobacteria [15]. Alterations in the relative abundance of these bacterial phyla have been associated with different pathologic conditions, such as Crohn’s disease, irritable bowel syndrome, and NAFLD [18]. In NAFLD, the dysbiosis is characterized by a reduction in microbial diversity, with a greater relative abundance of species of the Proteobacteria and Bacteroidetes phyla, the Enterobacteriaceae family and the *Escherichia* genera [19,20], and a lower abundance of the Firmicutes phylum and the Prevotellaceae family [19,21]. These changes have been linked to the progression of NAFLD by inducing inflammation through: (i) increased lipopolysaccharide (LPS) translocation; (ii) reduced short-chain fatty acids (SCFA) production in the GM; and (iii) increased endogenous ethanol production [10].

The augmentation of gram-negative bacteria in the GM increases the intestinal and hepatic exposure to LPS, a bacterial endotoxin that is part of the outer membrane [22]. On the other hand, the reduction of SCFA in this microbial community affects tight junctions, increasing the permeability of the intestinal barrier [23] and favoring bacterial translocation and hepatic exposure to LPS [24]. In the liver, LPS activates the innate immune system via Toll-like receptor 4 (TLR4) stimulation. LPS binds to CD14, activating TLR4 and the nuclear factor kappa-light-chain-enhancer of activated B cells (NFκB). This induces the expression of inflammatory cytokines such as tumor necrosis factor α (TNF-α) and interleukin 6 (IL-6), contributing to inflammation and insulin resistance, which can increase fat accumulation in the liver [25]. In addition, dysbiosis contributes to NAFLD pathogenesis due to an increase of endogenous alcohol synthesis, *de novo* lipogenesis (DNL), liver accumulation of triglycerides (TG), and decreased β-oxidation and choline levels [21].

Currently, there are no direct pharmacological treatments for NAFLD. Lifestyle changes, weight loss, and the use of drugs to reduce insulin resistance, such as pioglitazone, and vitamin E, due to its antioxidant effect for patients with biopsy-proven steatohepatitis [3], are the main strategies to control its progression. Nevertheless, over the last few years, different approaches targeting the GM have emerged based on its role in NAFLD pathogenesis. These strategies include probiotics, prebiotics, and synbiotics and aim to reverse microbial dysbiosis in the GM and reduce biological markers associated with NAFLD. In this scenario, these microbiota-focused treatments are of great interest for the prevention and treatment of NAFLD, and have gained attention during the past decade. This review analyzes the main effects of probiotics, prebiotics, and synbiotics on gut microbiota, liver damage, anthropometric parameters, blood lipids, markers of inflammation, and insulin resistance as a strategy for non-alcoholic fatty liver disease.

To assess the aim of this narrative review, a literature search of clinical trials published in English from 2015 to 2020 was performed in Web of Science, Scopus, Science Direct, and PubMed databases, with the following keywords: “non-alcoholic fatty liver disease” and “probiotics”, “prebiotics”, and/or “synbiotics”. Inclusion criteria for these publications were: (i) patients ≥ 18 years old diagnosed with NAFLD; and (ii) interventions exclusively with probiotics, prebiotics, and synbiotics. Studies including pharmacological interventions and not focused on the study of NAFLD were excluded. Studies including specific food plans as dietary interventions were not included in this selection.

The main findings of this narrative review are presented in Table 1, including the type, composition, and dosage of the probiotic/prebiotic/symbiotic treatment, the characteristics of the studied population, and the main effects of the intervention. Studies that included dietary recommendations and interventions are detailed in this table.

## 2. Effects of Probiotics and Prebiotics on the Gut Microbiota

Microbiota-focused strategies for NAFLD are based on the use of probiotics and prebiotics for the modulation of the GM. According to the World Health Organization (WHO), probiotics are “live microorganisms which when administered in adequate amounts confer a health benefit on the host” [44]. These health benefits include the improvement in barrier function, intestinal stimulation of the immune system, and protection against pathogens [45]. For this to occur, microorganisms must be capable of staying viable and in sufficient quantities upon reaching the intestine.

Currently, the main studied bacterial probiotic genera are *Lactobacillus* and *Bifidobacterium*, which can be found in different foods and supplements. In NAFLD, the effect of probiotics on the GM composition has been mainly evaluated using lactic acid bacteria belonging to these genera [46] (Table 1); nevertheless, different outcomes have been observed depending on the probiotic product and the dosage. Ahn and co-workers observed that supplementation for 12 weeks with a probiotic mixture including *Lactobacillus*, *Pediococcus*, and *Bifidobacterium* increased the relative abundance of microorganisms from these genera in the GM, specifically, *L. acidophilus*, *L. rhamnosus*, *P. pentosaceus*, *B. lactis*, and *B. brevis* species [26]. On the other hand, the consumption of conventional yogurt fermented by cultured *L. delbrueckii* subsp. *bulgaricus* and *Streptococcus thermophilus* decreased the relative abundance of the Firmicutes phylum, Clostridia, and Erysipelotrichia classes, Clostridiales and Erysipelotrichales orders, Erysipelotrichaceae and Veillonellaceae families, and *Blautia*, *Pseudobutyrivibrio*, *Eubacterium ventriosum*, *Ruminococcus*, and *Dialister* genera, while augmenting the Negativicutes class, Selenomonadales order, Acidaminococcaceae family, and *Phascolarctobacterium* genus [27].

Another implemented strategy to modulate GM is the use of prebiotics and synbiotics. Prebiotics are defined as “nonviable food components that confer a health benefit on the host associated with the modulation of the microbiota” [47]. These consist mainly of non-starch polysaccharides and oligosaccharides, which stimulate the growth of beneficial bacteria [48]. On the other hand, synbiotics are a mixture of probiotics and prebiotics. In patients with NAFLD, the consumption of fructooligosaccharides (FOS) in different doses and time periods (8 g/day for 12 weeks, and 16 g/day for 24 weeks) increased the relative abundance of the Actinobacteria phylum and *Bifidobacterium* genus in the GM and reduced the *Clostridium* genus [33] (Table 1).

The effect of synbiotics on the GM of patients with NAFLD has been evaluated using different lactic acid bacteria supplemented with FOS (Table 1). According to Scorletti and co-workers, concomitant treatment with *B. animalis* subsp. *lactis* BB-12 and FOS for 10–14 months produced an increase of the Actinobacteria and Firmicutes phyla. At the genus level, this treatment augmented the relative abundance of *Bifidobacterium* and *Faecalibacterium*, and reduced *Oscillibacter* and *Alistipes* [12]. On the other hand, the use of a synbiotic mixture composed by FOS and species of *Lactobacillus*, *B. longum*, and *S. thermophilus* for 12 weeks showed an increase in the relative abundance of different species of *Bifidobacterium* and *Lactobacillus*, and non-pathogenic strains of *E. coli* and *Enterococcus faecalis*. No changes in pathogenic strains of *E. coli* were observed with this treatment [34]. Altogether, these results show that probiotic-based strategies do not only modify the abundance of probiotic strains in the GM, but also induce changes in other bacterial taxa, reverting NAFLD dysbiosis.

NAFLD-associated dysbiosis is also characterized by an increase in gram-negative bacteria and LPS translocation to systemic circulation [22,24]. Bacterial LPS acts as a toxin at a systemic level and induces endotoxemia and inflammation [46], which contributes to NAFLD pathogenesis [10]. In this context, a reduction in this parameter is expected in microbiota-modifying treatments. Nevertheless, the consumption of probiotics, prebiotics, and synbiotics showed mostly no effect on LPS concentration in blood, regardless of the dose and the duration of the treatment [12,26,33]. Only two studies reported a significant decrease in this endotoxin concentration. Both studies evaluated the consumption of probiotics through different approaches: a probiotic yogurt [27] and a multi-strain probiotic [28] (Table 1). The main difference between these two treatments and the other evaluated products is related to the presence of *L. delbrueckii* subsp. *bulgaricus* and *S. thermophilus* in the probiotic mixture, suggesting that the effect of probiotic-based strategies over LPS concentration is species-dependent.

Data obtained from these clinical trials agree with pre-clinical studies, where consumption of probiotics (*Lactobacillus* and *Bifidobacterium*) modulates the GM and reduces LPS concentration, ameliorating the NAFLD-dysbiosis in rats [49,50,51].

## 3. Effect of Probiotics, Prebiotics, and Synbiotics on Liver Damage

At first, NAFLD diagnosis is based on different clinical biomarkers such as liver enzymes, whose moderate or slight elevation is associated with liver injury. While an increase in aspartate aminotransferase (AST) and alanine aminotransferase (ALT) levels is associated to liver damage, gamma-glutamyl-transpeptidase (GGT) and alkaline phosphatase (ALP) are linked to altered liver excretion [52]. In this scenario, different studies have explored the effects of prebiotics, probiotics, and synbiotics on liver damage (Table 1), focusing mainly on the use of different species of *Lactobacillus*, *Bifidobacterium*, and *Streptococcus* as probiotics, and FOS as prebiotic. Prebiotic and probiotic supplementation in patients with liver damage has shown to significantly decrease AST [28,29,34,36,37,38,39,40,42] and ALT levels [27,28,34,36,37,38,39,40,42], and this effect was independent of the treatment duration (>8 weeks) and probiotic/prebiotic dosage.

Regarding serum concentrations of GGT, synbiotic supplementation produced a significant reduction in this parameter [36,39]; similar reductions were seen with multi-strain probiotic supplementation (*Lactobacillus*, *Lactococcus*, *Bifidobacterium*, *Propionibacterium*, and *Acetobacter*) [29]. On the contrary, the intake of FOS as prebiotic for 9 months did not produce significant changes in GGT [33]. 

On the other hand, serum ALP levels were modified with the use of probiotics, prebiotics, and synbiotics in all studies where ALP was measured [28,36,37,38,40], including treatments with a multi-strain probiotic (different species of *Lactobacillus*, *Bifidobacterium*, and *S. thermophilus*) for 12 months [28], a synbiotic yogurt (*B. animalis* ssp. *lactis* (BB-12) + 1.5 g of inulin) [36], and a synbiotic mixture (different species of *Lactobacillus*, *Bifidobacterium*, and *S. thermophilus* + FOS or inulin) [37,38,40].

Because liver enzymes levels are not specific to NAFLD [53], liver biopsy is the gold standard for differential diagnosis. Nevertheless, it is not frequently performed because it is an invasive and high-cost method. In this context, less invasive and less expensive imaging studies are mainly used to evaluate fibrosis and steatosis, such as liver ultrasound or nuclear magnetic resonance, respectively [54]. In this context, treatment with probiotics and synbiotics has shown to reduce hepatic fibrosis in patients with steatohepatitis [28,39] (Table 1). In these cases, the use of probiotics (*Lactobacillus*, *Bifidobacterium*, and *S. thermophilus*) and synbiotics (*Lactobacillus*, *Bifidobacterium*, *S. thermophilus*, and FOS) showed the same effect [28,39]. On the contrary, a clinical trial using *B. animalis* subsp. *lactis* and FOS as synbiotic did not observed differences in this parameter [12]. These differences suggest that the effect of synbiotics over fibrosis depends on the probiotic strains, regardless of the prebiotic.

The use of prebiotics and synbiotics has had significant effects in decreasing the degree of hepatic steatosis (hepatic fat infiltration) in patients with NASH [33,35,43] (Table 1). In this scenario, Bomhof and co-workers observed that FOS supplementation decreased steatosis and overall NAS [33]. Ferolla and colleagues reported that after synbiotic supplementation with *L. reuteri*, guar gum, and inulin, the proportion of patients with moderate/severe steatosis decreased from 40.7% to 18.5%, increasing patients with mild steatosis from 59.2% to 81.5% [35]. Another study carried out by Asgharian and co-workers showed that synbiotic treatment with different species of *Lactobacillus*, *Bifidobacterium*, and *Streptococcus thermophilus* and FOS reduced steatosis in NAFLD. In this work, 50% of patients with mild steatosis became normal, 25% of those with moderate steatosis became normal, and 43.8% of patients with moderate steatosis became mild [43].

While Ferolla reported a positive effect in this matter with the usage of a synbiotic containing inulin and guar gum for 3 months [35], Chambers and co-workers did not report changes in steatosis in patients treated with 20 g/day of inulin or inulin propionate for 42 days [55]. Besides the complementary effect of prebiotics and probiotics in Ferolla’s study, it cannot be ruled out that these differences are due to the exposure periods.

These findings support the results obtained in studies with animal models, indicating that consumption of probiotic strains reduces liver damage in NAFLD [56,57,58] as well as prebiotics and synbiotics [51,59]. Hepatoprotective effects of probiotic lactic acid bacteria belonging to *Lactobacillus* and *Bifidobacterium* have been previously reported in animal models, where the effect of these microorganisms has been associated with the inhibition of ß-glucuronidase [60] and the reduction of Gpr109a SCFA receptor in liver and adipose tissue [61]. The reduction of liver fibrosis due to probiotic treatment (*L. rhamnosus*) has been associated with the inhibition of hepatic bile salts biosynthesis and the enhancement of their excretion in animal models [62], while the effect of probiotic consumption on liver steatosis has been linked to an increase in hepatic Natural Killer T-cells (NKT) and reduced inflammatory signaling [63], and to bacteria and host competition for fatty acids absorption [58]. On the other hand, prebiotics have shown to ameliorate liver damage through the suppression of the LPS-TLR4-Mψ axis, secondary to GM modulation [64], and the interventions with FOS have demonstrated to reduce hepatic steatosis due to the induction of gene expression in the liver [65].

## 4. Effect of Probiotics, Prebiotics, and Synbiotics on Anthropometric Parameters

Patients with NAFLD with non-alcoholic steatohepatitis (NASH) present alterations of the energy homeostasis and increased systemic inflammation by means of diverse mechanisms. This leads to a reduction in mitochondrial fatty acid oxidation, ketogenesis, glucose uptake, and insulin secretion [66]. In addition, it produces an increase in lipogenesis and cholesterol and triglyceride biosynthesis, promoting weight gain [66]. Several studies have shown positive results in body composition with the use of probiotics, prebiotics, and synbiotics (Table 1). The use of a probiotic mixture (*Lactobacillus*, *Bifidobacterium*, and *P. pentosaceus*) [26], probiotic yogurts with *Lactobacillus* and *Streptococcus* [27], and *B. lactis* [30] significantly decreased body weight, body mass index (BMI), and waist circumference (WC) in patients with NAFLD. The same effect was reported with synbiotic treatments consisting of strains of *Lactobacillus*, *Bifidobacterium*, and *Streptococcus* plus FOS, inulin or guar gum with inulin [34,35,41,42]. On the other hand, a significant reduction in fatty liver index, intrahepatic fat fraction, body fat, and visceral fat was also observed after 8–12 weeks of supplementation with a probiotic mixture [26,29]. Body fat reduction was observed with the use of a probiotic yogurt [27] and a synbiotic [41], and decreases in the grade of fatty liver were detected after 12–24 weeks prebiotic supplementation with inulin and probiotic strains of *L. acidophilus* and *Bifidobacterium* [36,40]. In contrast to these results, Bomhof and co-workers detected that a 12-week prebiotic supplementation with FOS did not affect body composition [33]. In this scenario, the effect of probiotics and synbiotics on anthropometric parameters seems to be directly related to the microbial component. In the study of Ahn et al., decreased body weight was linked to changes in the GM due to the consumption of probiotics, being positively associated to some species of *Dorea* [26].

In pre-clinical studies, probiotic treatments have shown to improve anthropometric parameters that are altered in NAFLD, such as weight. Specifically, different species of *Lactobacillus* and *Bifidobacterium* have shown to reduce body weight and body weight gain in high-fat fed animals [61,67,68]. Thus, the outcomes observed from clinical trials agree with pre-clinical reports, and the mechanisms involved in this effect have been associated to the remodeling of energy metabolism [69,70]. Regarding prebiotic treatments, high-prebiotic diets (inulin + oligofructose) can increase satiety hormone levels (glucagon-like peptide 1 and peptide-YY), reducing food intake [71].

## 5. Effect of Probiotics, Prebiotics, and Synbiotics on Blood Lipids

Patients with NAFLD present high levels of blood lipids, due to alterations in their metabolism. It has been observed that supplementation with probiotics composed of multiple bacterial strains, mainly formed by *Lactobacillus*, *Bifidobacterium*, and *Propionibacterium* species, for 8 and 12 weeks, produces a decrease in triglycerides (TG) and total cholesterol (TC) [26,29] (Table 1). After supplementation with yogurt for 24 weeks, decreases in TG y TC have also been detected [27]. Furthermore, probiotic supplementation with multiple strains produced a significant decrease in low-density cholesterol (LDL-C) [29].

Regarding the use of synbiotics, a study evaluating 300 g of synbiotic yogurt with 10^8^ CFU/day of *Bifidobacterium* as probiotic and inulin for 24 weeks reported a significant decrease in serum concentrations of TC, TG, and LDL-C [36]. A decrease in the same parameters was observed after an 8-weeks supplementation with a synbiotic composed by different strains of *Lactobacillus*, *Bifidobacterium*, and *Streptococcus* as probiotics and FOS [37]. Additionally, studies based on the use of strains of *Lactobacillus*, *Bifidobacterium*, and *Streptococcus* as probiotics and FOS as a prebiotic, for a period between 8 and 12 weeks, have also shown to significantly reduce TC [34,41] and LDL-C [41]. According to the above, the effect of microbial-based strategies on blood lipids is related to the probiotic microorganisms, in a strain-specific manner, as reported by Xie et al. [72].

In pre-clinical studies, an improvement in blood lipids has been reported after probiotic and prebiotic treatments [51,61,67,68,73,74]. Reduction in TC due to single and combined probiotic treatment (*Lactobacillus*) has been linked to the activation of the transcription of genes belonging to the liver X receptors (LXR) axis, inducing TC reverse transport and augmenting the conversion of TC to bile acids [75]. On the other hand, the underlying mechanism for the reduction of TG levels in this study was the inhibition of transcription genes of carbohydrate reaction element binding protein and the activation of the transcription of genes encoding the peroxisome proliferator-activated receptor alpha (PPARα) [75]. It is important to notice that, as well as in clinical trials, the effect of these bacterial genus on lipid metabolism has shown to be species-specific in animals [76].

## 6. Effect of Probiotics, Prebiotics, and Synbiotics on Inflammation Markers

In NAFLD, there is an increase in the production and release of pro-inflammatory cytokines due to lipotoxicity and insulin resistance, among other factors [10]. Therefore, it is of special interest to evaluate the effect of probiotics, prebiotics, and synbiotics on these biomarkers. Supplementation with multi-strain probiotics (*Lactobacillus*, *Bifidobacterium*, *S. thermophilus*, *Propionibacterium*, and *Acetobacter)* has shown to reduce IL-6, TNF-α [28,29,30,31], and IL-1β levels in these patients [28]. Additionally, Chen and co-workers observed a significant decrease in TNF-α levels after the consumption of yogurt containing *L. delbrueckii* subp. *bulgaricus* and *S. thermophiles* for 24 weeks [27]. Supplementation with a synbiotic treatment composed of strains of *Lactobacillus*, *Bifidobacterium*, *S. thermophilus*, and FOS for 8 weeks also produced a significant decrease in TNF-α levels [38]. Changes are also observed in TNF-α, but not in IL-6, after synbiotic [42], probiotic [26,42], and prebiotic [42] supplementation. In this scenario, the impact of probiotic/synbiotic supplementation seems to be highly related to the bacterial strains than to other variables such as intervention duration.

In pre-clinical trials, consumption of probiotics from different genera, such as *Lactobacillus* and *Bifidobacterium*, and prebiotics has shown to reduce inflammation in rats with NAFLD [49,51,57], and changes in this parameter have been associated with the modulation of the GM [49,51]. Additionally, synbiotic treatment has also been linked to a reduction in the expression of pro-inflammatory cytokines in animal models [59].

## 7. Effect of Probiotics and Synbiotics on Insulin Resistance

Insulin resistance is one of the main factors involved in the development and progression of NAFLD [10]. In this context, Sepideh and co-workers observed a significant decrease in fasting glucose, insulin, and HOMA-IR after supplementation of multi-strain probiotics (*Lactobacillus*, *Bifidobacterium*, and *S. thermophilus)* [31]. Chen and co-workers also observed significant changes in HOMA-IR levels and fasting insulin and showed a decreasing trend in fasting blood sugar (FBS) after consumption of 220 g of yogurt fermented with *L. delbrueckii* subsp. *bulgaricus* and *S. thermophilus* for 24 weeks [27]. On the other hand, insulin, HOMA-IR, and glucose levels did not change after 12 weeks of consumption of a probiotic mixture (*Lactobacillus*, *Bifidobacterium*, and *P. pentosaceus*) [26]. Nevertheless, Nabavi et al. did not observe changes after the consumption of 300 g of a probiotic yogurt produced by strains of *Lactobacillus*, *B. lactis*, and *S. thermophilus* compared to the consumption of a conventional yogurt for 8 weeks [30]. These differences could be due to changes in intervention periods and probiotic doses between studies.

Regarding the use of synbiotics, a significant decrease in fasting glucose [37,39] and insulin levels [37] was observed after supplementation with *Lactobacillus*, *Bifidobacterium*, *Streptococcus thermophilus*, and FOS for 8 and 28 weeks [37,39].

The analyzed clinical studies agree with prior results obtained from animal models indicating that the treatment with probiotic and synbiotics enhanced insulin resistance in NAFLD, measured through fasting glucose, post-prandial glucose, and/or insulin levels [51,57,59,61,73]. The antidiabetic effect of *Lactobacillus* is associated with the modulation of the GM, the increase in short-chain fatty acids-producing bacteria, and the modification of liver gene expression, improving glucose metabolism [68,77].

## 8. Safety and Tolerability of Probiotics and Prebiotics for Treatment of NAFLD

Probiotics have been safely used in foods and fermented products for hundreds of years and several probiotic strains, such as *B. lactis* and *S. thermophilus*, are currently categorized as generally recognized as safe (GRAS) by the US Food and Drug Administration (FDA) [78]. In 2011, the Agency for Healthcare Research and Quality (AHRQ) reviewed the safety of probiotic interventions for the prophylaxis and treatment of diseases [79]. This report indicated that probiotics belonging to *Lactobacillus*, *Bifidobacterium*, *Saccharomyces*, *Streptococcus*, *Enterococcus*, and/or *Bacillus* genera did not increase health risks [80]. However, one of the main limitations to assess the safety and tolerability of probiotic interventions is the lack of information on this matter in randomized controlled trials (RCTs). In general terms, probiotic-associated side effects include gastrointestinal symptoms, bacteremia, gene transfer of antibiotic resistance determinants, metabolic alterations, and systemic infections. Nevertheless, these rarely appear in the literature [79]. Regarding NAFLD, there is a lack of reports of adverse effects in clinical trials; therefore, it is of great relevance to address this issue in future studies.

Prebiotics (inulin and FOS) have the generally recognized as safe (GRAS) status in the United States and are considered as natural food ingredients in most European countries [81]. While there are a few concerns with prebiotic supplementation [82], Kaur and Gupta [83] reported that high doses of these compounds (30 g/d) may cause adverse gastrointestinal effects, mainly flatulence. It has been demonstrated that prebiotics consumption has beneficial effects over human health through the modulation of the GM [84,85,86], being safe and well tolerated [87,88].

## 9. Pharmacological and Microbiota-Based Strategies for NAFLD

While lifestyle interventions, such as exercise and eating habits, remain the first-line strategy for NAFLD management, currently, different pharmacological approaches are being implemented to prevent and revert this illness and delay its progress. These strategies are mainly focused on comorbidities such as obesity, diabetes, and lipid disorders, aiming to reduce IR, inflammation, and oxidative stress [3]. Therapeutic trials have demonstrated that antidiabetic drugs such as liraglutide, pioglitazone, metformin, and a glucagon-like peptide 1 (GLP-1) might help to reduce hepatic steatosis [89,90,91] and liver fibrosis [89,90,92,93], while reducing NAFLD [94]. On the other hand, lipid profile-modifying compounds have not shown significant effect on liver histopathology [95,96]. 

The effects of anti-diabetic drugs, mainly metformin, on the GM in NAFLD-associated dysbiosis have been studied with positives outcomes [97]. Thus, these pharmacological agents have become a potential gut-based treatment, although no hepatic histological benefit have been reported [94]. While numerous drugs are currently under investigation for NAFLD management [98], there is still no specific pharmacologic treatment approved by the FDA or the European Medicines Agency [99].

Microbiota-based therapeutics, such as probiotic, prebiotic, and synbiotic supplementation, have demonstrated a significant impact on GM modulation as well as beneficial effects in NAFLD treatment. Probiotics efficiently reduced NAFLD dysbiosis, fatty acid synthesis, inflammation, and metabolic endotoxemia in animal models [100,101,102], as well as liver aminotransferases, serum pro-inflammatory cytokine levels, total cholesterol and triglycerides, insulin sensitivity, HOMA-IR, and BMI in human interventions (Table 1). Prebiotics have proven to affect carbohydrate and lipid metabolism by decreasing insulin, glucose, triglyceride, and cholesterol levels as well as transaminase activity [103,104,105]. Synbiotics have also shown GM modulation properties, as well as lowering levels of cholesterol, transaminase activity and, similar to probiotics, pro-inflammatory cytokines, in adults and murine models [59].

Since there is no specific pharmacological treatment approved for NAFLD and probiotics, prebiotics, and synbiotics have shown a beneficial impact not only in reverting dysbiosis, but also in clinical markers of the disease in several pre-clinic and clinic studies, the use of this bioactive compounds for the prevention of NAFLD or as a complementary strategy for its treatment appears to be of great interest.

## 10. Probiotics, Prebiotics, and Synbiotics as a Complementary Strategy to Specific Food Plans as Dietary Interventions for NAFLD

One of the main factors modulating the gut microbiota is diet, where high-fat/high-carbohydrates/low-fiber diets are known to induce gut dysbiosis characterized by higher relative abundance of the phylum Proteobacteria, genus *Bacteroides* [106], classes Erysipelotrichia and Gammaproteobacteria [107], including ethanol-producing *E. coli* [108], and lower abundance of bacteria belonging to phylum Firmicutes such as *Ruminococcus bromii* and *Roseburia* [109], favoring a pro-inflammatory state. On the other hand, fiber-rich and high mono- and poly-unsaturated fatty acids diets are known to promote a healthy microbiota [110]. Therefore, due to the role of GM on NAFLD and the impact of diet on this microbial community, dietary interventions have been proposed as complementary strategies for NAFLD. Among these dietary patterns, the Mediterranean diet and vegetarian/vegan diets have shown to improve gut microbiota dysbiosis, augmenting *Bifidobacterium* [111], *Prevotella* [112], and *Faecalibacterium prausnitzii* [113], while reducing *E. coli* [111] and other gram-negative bacteria [114]. This changes in GM have been linked to a reduction in intestinal permeability, LPS levels, and metabolic endotoxemia [115], which may be beneficial in NAFLD [116], as well as lowering weight and inflammation [111,117] and stimulating adiponectin secretion that is associated with NAFLD alleviation [118]. In this context, because of the effects of probiotics, prebiotics, and synbiotics on NAFLD evolution, their association with changes in dietary patterns could be of great interest as a new complementary approach in this pathology treatment. In this scenario, further studies are needed to evaluate if the combination of these strategies represents an advantage and potentiates the effects of each other.

## 11. Future Challenges in the Study of the Usage of Probiotics, Prebiotics, and Synbiotics for NAFLD

Despite several studies addressing the effects of the treatment with probiotics, prebiotics, and synbiotics for NAFLD on humans are currently available, the main limitations of these clinical trials are related to the number of participants and the differences in dosage and duration of the treatments. Additionally, in most of the studies, the response to probiotic, prebiotic, and synbiotic intake was not evaluated by liver biopsy. On the other hand, differences in the administration forms (powder, capsules, and yogurt) can also produce differences in the clinical outcome due to microbial viability in the gastrointestinal tract. Another issue that should be addressed is that most analyzed studies evaluated the effect of this microbiota-targeting strategies on clinical biomarkers associated with NAFLD; nevertheless, the effect of these compounds over the composition and metabolism of GM has not been fully addressed. Furthermore, regarding GM studies, the co-occurrence of other metabolic disorders known to produce dysbiosis, such as obesity and type 2 diabetes, in patients with NAFLD may interfere in the clinical outcomes of these strategies.

In this context, future studies on this field should aim to increase the number of volunteers enrolled in the clinical trials. Additionally, most of the studies have been performed in the Asian region; thus, it is of great importance to incorporate other geographical areas to the analyses because of their possible differences in GM and response to the above-mentioned treatments. On the other hand, the incorporation of metagenomic and metabolomic approaches to evaluate the effects of probiotics, prebiotics, and synbiotics on the GM in NAFLD will contribute to elucidate the mechanisms of action of these strategies.

## 12. Conclusions

The use of probiotics, prebiotics, and synbiotics has emerged as a new strategy for the treatment of non-alcoholic fatty liver disease (NAFLD). Evidence shows that the use of these gut microbiota-focused treatments reverts gut dysbiosis associated with NAFLD, enhancing biomarkers of the disease. This strategy reduces liver damage, inflammation, and insulin resistance associated with NAFLD. The use of this therapeutic approach also enhances body weight and blood lipids. Altogether, these results show a beneficial effect of probiotics, prebiotics, and synbiotics over NAFLD and their effect depend on the type of treatment, dosage, and exposure period. Finally, to fully comprehend the effect of microbiota-based strategies on the evolution of NAFLD, further studies are needed.

## Figures and Tables

**Table 1:** Main effects of probiotic, prebiotic, and synbiotic treatments on patients with NAFLD.

Type of Treatment	Characterization of the Studied Population/Dietary Recommendations	Composition	Dosage	Effects	Ref.
Probiotic	*n* = 65 m/f ratio = 1.03 Patients with BMI > 25 kg/m^2^ diagnosed with NAFLD, between 19 to 75 years old. The patients were educated on appropriate daily nutritional intake and exercise.	*L. acidophilus* CBT LA1 *L. rhamnosus* CBT LR5 *L. paracasei* CBT LPC5 *P. pentosaceus* CBT SL4 *B. lactis* CBT BL3 *B. breve* CBT BR3	10^9^ CFU/day of probiotic strains, for 12 weeks.	↑ *L. acidophilus*, *L. rhamnosus*, *P. pentosaceus*, *B. lactis* and *B. breve* ↑ *Agathobaculum*, *Dorea* (OTU 527923), *Dorea* (OTU 195044), *Blautia*, *Ruminococcus*, and *Dorea* (OTU 470168). ↓ Intrahepatic fat fraction ↓ TC, TG ↓ BMI, weight ↓ Total fat mass, % total body fat and visceral fat ↓ TNF-α	[26]
	*n* = 92 Only female patients with BMI ≥ 28 kg/m^2^ diagnosed with NAFLD and MetS, between 36 and 66 years old.	*L. delbrueckii* subsp. *bulgaricus* *S. thermophilus*	220 g of yogurt/day, for 24 weeks.	↓ Firmicutes, Clostridia and Erysipelotrichia ↓ Clostridiales and Erysipelotrichales ↓ Erysipelotrichaceae and Veillonellaceae ↓ *Blautia*, *Pseudobutyrivibrio*, *Eubacterium ventriosum*, *Ruminococcus* and *Dialister* ↑ Negativicutes, Selenomonadales, Acidaminococcaceae and *Phascolarctobacterium*. ↓ LPS ↓ ALT ↓ Fat mass ↓ TC, TG ↓ FBS ↓ Fasting insulin, HOMA-IR ↓ TNF-α	[27]
	*n* = 3 m/f ratio = n.s. Patients diagnosed with NAFLD, ≥18 years old. The patients were advised to follow a healthy lifestyle, and patients with overweight/obesity were advised to follow a hypocaloric diet (30% reduction in calorie intake).	*L. paracasei* DSM 24733 *L. plantarum* DSM 24730 *L. acidophilus* DSM 24735 *L. delbrueckii* subsp. *bulgaricus* DSM 24734 *B. longum* DSM 24736 *B. infantis* DSM 24737 *B. breve* DSM 24732 *S. thermophilus* DSM 24731	2 × 10^11^ CFU/day of probiotic strains, for 12 months.	↓ Endotoxins ↓ NAS ↓ Steatohepatitis ↓ ALP, AST, ALT ↓ TNF-α, IL-1β, IL-6	[28]
	*n* = 58 m/f ratio n.s. Patients with BMI > 25 kg/m^2^ diagnosed with NAFLD and type 2 diabetes, between 18 and 65 years old. The patients were advised to follow a healthy lifestyle and to continue with their antihyperglycemic treatment.	*Lactobacillus* *Lactococcus* *Bifidobacterium* *Propionibacterium* *Acetobacter*	6 × 10^10^ CFU/day of *Lactobacillus* and *Lactococcus* + 1 × 10^10^ CFU/day of *Bifidobacterium* + 3 × 10^10^ CFU/day of *Propionibacterium* + 1 × 10^6^ CFU/day of *Acetobacter*, for 8 weeks.	↓ Fatty Liver Index ↓ AST, GGT ↓ TC, TG, LDL-C ↓ TNF-α, IL-6	[29]
	*n* = 72 m/f ratio = 0.95 Patients with BMI between 25 and 40 kg/m^2^ diagnosed with NASH, between 23 and 40 years old.	*L. bulgaricus* *L. acidophilus* *B. lactis* *S. thermophilus*	300 g of yogurt/day of probiotic strains, for 8 weeks.	↓ BMI, weight	[30]
	*n* = 42 m/f ratio = 2.00 Patients diagnosed with NAFLD, between 18 and 65 years old.	*L. casei* *L. acidophilus* *L. rhamnosus* *L. bulgaricus* *B. breve* *B. longum* *S. thermophilus*	3 × 10^9^ CFU/day of *L. casei* + 3 × 10^10^ CFU/day of *L. acidophilus* + 7 × 10^9^ CFU/day of *L. rhamnosus* + 5 × 10^8^ CFU/day of *L. bulgaricus* + 2 × 10^10^ CFU/day of *B. breve* + 1 × 10^9^ CFU/day of *B. longum* + 3 × 10^8^ CFU/day of *S. thermophilus*, for 8 weeks.	↓ TNF-α, IL-6 ↓ FBS ↓ Insulin, HOMA-IR	[31]
Prebiotic	*n* = 60 m/f ratio = n.s. Patients with BMI > 27 kg/m^2^ diagnosed with NAFLD and type 2 diabetes or MetS, between 18 to 75 years old. The patients were advised to follow a healthy lifestyle and diet.	Inulin	4 g of inulin/day, for 4 weeks	↓ ALT	[32]
	*n* = 14 m/f ratio = 1.00 Patients with BMI > 23 kg/m^2^ diagnosed with NASH, ≥18 years old.	FOS	8 g of FOS/day, for 12 weeks, then, 16 g/day, for 24 weeks.	↑ *Bifidobacterium* ↓ *Clostridium* cluster XI and I ↓ Hepatic steatosis ↓ NAS	[33]
Synbiotic	*n* = 89 m/f = 1.90 Patients diagnosed with NAFLD.	FOS *B. animalis* subsp. *lactis* BB-12	8 g of FOS/day + 10^9^ CFU/day of probiotic strain, for 10–14 months.	↑ *Bifidobacterium* and *Faecalibacterium* ↓ *Oscillibacter* and *Alistipes*	[12]
	*n* = 75 m/f ratio = 0.56 Patients diagnosed with NASH, ≥18 years old. Low-fat/low-calorie food plan	FOS *L. casei* *L. rhamnosus* *L. bulgaris* *B. longum* *S. thermophilus*	FOS (n.s.) + 10^8^ CFU/day of probiotic strains, for 12 weeks.	↑ *Bifidobacterium*, *Lactobacillus*, non-pathogenic *E. coli* and *Enterococcus faecalis* ↓ ALT, AST ↓ TC ↓ BMI	[34]
	*n* = 50 m/f ratio = 0.58 Patients diagnosed with NASH, between 25 to 74 years old. Food plan of 1500 kcal/day for women and 1800 kcal/day for men	Guar gum Inulin *L. reuteri*	8 g of partially hydrolyzed guar gum and inulin/day + 2 × 10^8^ CFU/day of probiotic strain, for 3 months.	↑ LPS ↓ Hepatic steatosis ↓ BMI, WC	[35]
	*n* = 102 m/f ratio = 0.96 Patients diagnosed with NAFLD, ≥18 years old. The patients were advised to follow a healthy lifestyle.	Inulin *Bifidobacterium animalis* subsp. *lactis* (BB-12)	1.5 g inulin/day + 300 g of yogurt supplemented with 10^8^ CFU/day of probiotic strain, for 24 weeks.	↓ Grade of fatty liver ↓ AST, ALT, ALP, GGT ↓ TC, TG, LDL-C	[36]
	*n* = 60 m/f ratio = 4.00 Patients with BMI between 25 and 35 kg/m^2^ diagnosed with NAFLD, between 25 and 64 years old.	FOS *L. casei* *L. rhamnosus* *L. acidophilus* *L. bulgaricus* *B. breve* *B. longum* *S. thermophilus*	FOS (n.s.) + 4 × 10^8^ CFU/day of probiotic strains, for 8 weeks.	↓ ALT, AST, ALP ↓ TC, TG, LDL-C ↓ FBS, Insulin	[37]
	*n* = 60 m/f ratio = 4.00 Patients with BMI between 25 and 35 kg/m^2^ diagnosed with NAFLD, between 25 and 64 years old.	FOS *L. casei* *L. rhamnosus* *L. acidophilus* *L. bulgaricus* *B. breve* *B. longum* *S. thermophilus*	FOS (n.s.) + 4 × 10^8^ CFU/day of probiotic strains, for 8 weeks.	↓ ALT, AST, ALP ↓ TNF-α	[38]
	*n* = 42 m/f ratio = 1.21 Patients with BMI ≤ 25 kg/m^2^ diagnosed with NAFLD, ≥18 years old. The patients were advised to follow a healthy lifestyle.	FOS *L. casei* *L. rhamnosus* *L. acidophilus* *L. bulgaricus* *B. breve* *B. longum* *S. thermophilus*	125 mg of FOS/day + 2 × 10^8^ CFU/day of probiotic strains, for 28 weeks	↓ Hepatic steatosis and fibrosis ↓ AST, ALT, GGT ↓ TNF-α, NF-κB ↓ FBS	[39]
	*n* = 75 m/f ratio = 4.00 Patients diagnosed with NAFLD, between 20 and 60 years old.	HP inulin *B. longum* *L. acidophilus*	10 g/day of HP inulin + 2 × 10^7^ CFU/day of probiotic strains, for 3 months	↓ Grade of fatty liver ↓ AST, ALT, ALP	[40]
	*n* = 74 m/f ratio = 0.35 Patients diagnosed with NAFLD, between 18 and 60 years old.	FOS *L. casei* *L. acidophilus* *L. rhamnosus* *L. bulgaricus* *B. breve* *B. longum* *S. thermophilus*	500 mg/day of the synbiotic mixture, for 8 weeks.	↓ TC, LDL-C ↓ Weight ↓ Fat mass	[41]
	*n* = 75 m/f ratio = 4.00 Patients diagnosed with NAFLD, between 20 and 60 years old.	Inulin *L. acidophilus* *B. longum*	10 g/day of inulin + 10^7^ CFU/day of probiotic strains, for 3 months.	↓ BMI ↓ WC ↓ ALT, AST ↓ TNF-α	[42]
	*n* = 74 m/f ratio = 0.35 Patients diagnosed with NAFLD, between 18 and 60 years old.	FOS *L. casei* *L. acidophilus* *L. rhamnosus* *L. bulgaricus* *B. breve* *B. longum* *S. thermophilus*	500 mg/day synbiotic mixture, for 8 weeks.	↓ Steatosis	[43]

CFU: colony forming units, TC: total cholesterol, TG: triglycerides, LPS: lipopolysaccharides, ALT: alanine aminotransferase, AST: aspartate aminotransferase, GGT: gamma-glutamyl transferase, ALP: alkaline phosphatase, BMI: body mass index, NAS: NAFLD activity score, TNF-α: tumor necrosis factor alpha, NF-κB: nuclear factor κB, IL-1β: interleukin 1β, IL-6: interleukin 6, LDL-C: low density lipoprotein–cholesterol, WC: waist circumference, FBS: fasting blood sugar, HOMA-IR: homeostatic model assessment–insulin resistance, BMI: body mass index, m/f ratio: male/female ratio, n.s.: not specified.
